# Redox/NIR dual-responsive MoS_2_ for synergetic chemo-photothermal therapy of cancer

**DOI:** 10.1186/s12951-019-0510-2

**Published:** 2019-07-03

**Authors:** Jian Liu, Feiyang Li, Junxia Zheng, Bifei Li, Doudou Zhang, Lee Jia

**Affiliations:** 1 Cancer Metastasis Alert and Fujian Provincial Key Laboratory of Cancer Metastasis Chemoprevention and Chemotherapy, Fuzhou University, 2 Xueyuan Road, Sunshine Technology Building, 6FL, Fuzhou, 350116 Fujian China; 2 Institute of Oceanography, Minjiang University, Fuzhou, 350108 Fujian China

**Keywords:** MoS_2_ nanosheets, HA, Disulfide linkage, Dual-stimuli-responsive drug release, Chemo-photothermal therapy

## Abstract

**Background:**

The construction of a multifunctional drug delivery system with a variety of advantageous features, including targeted delivery, controlled release and combined therapy, is highly attractive but remains a challenge.

**Results:**

In this study, we developed a MoS_2_-based hyaluronic acid (HA)-functionalized nanoplatform capable of achieving targeted delivery of camptothecin (CPT) and dual-stimuli-responsive drug release. HA was connected to MoS_2_ via a disulfide linkage, forming a sheddable HA shell on the surface of MoS_2_. This unique design not only effectively prevented the encapsulated CPT from randomly leaking during blood circulation but also significantly accelerated the drug release in response to tumor-associated glutathione (GSH). Moreover, the MoS_2_-based generated heat upon near-infrared (NIR) irradiation could further increase the drug release rate as well as induce photothermal ablation of cancer cells. The results of in vitro and in vivo experiments revealed that MoS_2_–SS–HA–CPT effectively suppressed cell proliferation and inhibited tumor growth in lung cancer cell-bearing mice under NIR irradiation via synergetic chemo-photothermal therapy.

**Conclusions:**

The as-prepared MoS_2_–SS–HA–CPT with high targeting ability, dual-stimuli-responsive drug release, and synergistic chemo-photothermal therapy may provide a new strategy for cancer therapy.

**Electronic supplementary material:**

The online version of this article (10.1186/s12951-019-0510-2) contains supplementary material, which is available to authorized users.

## Introduction

Cancer has already been proven to be a major threat to human health, with more than eight million cancer-related deaths each year worldwide [1]. Chemotherapy is still the most common approach to cancer therapy, and approximately 80 different anticancer drugs are used in clinical treatment [2]. Unfortunately, these conventional anticancer drugs suffer numerous limitations, including drug resistance, nonspecific distribution, toxic side effects, and hydrophobicity [3, 4]. Therefore, numerous nanosized drug delivery systems with unique properties, such as active targeting, enhanced permeation and retention (EPR) effect, and stimuli-responsive drug release have been constructed to overcome these shortcomings [5–9]. In particular, multifunctional nanomaterials for combined therapy have the potential to realize great therapeutic effects on cancer, one of which is the ability to combine chemotherapy and photothermal therapy [10, 11]. As a minimally invasive or noninvasive approach to cancer treatment, photothermal therapy, which utilizes absorbed NIR light to generate hyperthermia for tumor ablation, has attracted widespread attention [12]. To date, several nanomaterials with strong NIR absorbance have been used as photothermal agents for cancer therapy, such as gold nanorods, CuS particles, carbon nanotubes, and reduced graphene [13–16]. More importantly, these nanomaterials are used to develop photothermally controlled drug release systems, which can reduce drug doses and avoid side effects. Therefore, integrating photothermal therapy and chemotherapy into one nanoplatform appears to be an effective therapeutic approach to cancer therapy.

Molybdenum disulfide (MoS_2_), a kind of two-dimensional (2D) transition metal dichalcogenide (TMDC), has been widely investigated for applications in electronic devices and catalysis because of its unique properties [17, 18]. Recently, several research teams have explored MoS_2_ for biomedical applications, particularly in drug and gene delivery [19]. Additionally, the high NIR absorbance of TMDCs has prompted research on photothermal therapy with MoS_2_ [20–29]. However, the application of MoS_2_ in biomedicine is limited because of its severe agglomeration in a physiological environment [21]. To address this problem, several polymers have been used to enhance the physiological stability of MoS_2_ [21, 29]. HA, a water-soluble mucopolysaccharide consisting of *N*-acetyl-d-glucosamine and d-glucuronic acid, exhibits excellent biodegradability, biocompatibility and nonimmunogenicity [30]. In addition, HA can specifically bind the cluster determinant 44 (CD44), which is overexpressed on the surface of various tumor cells [31, 32]. Because of the unique properties of HA, Dong et al. recently developed a HA-decorated MoS_2_ nanoplatform with high physiological stability as a drug carrier for the targeted delivery of doxorubicin [33]. Unfortunately, HA can form a shell on the surface of MoS_2_ nanosheets after HA coating, similar to graphene oxide (GO), and this shell can restrict the release of the loaded drug, which seriously reduces the therapeutic efficacy of the nanosheets [34]. To achieve rapid intracellular drug release, stimuli-responsive nanoplatforms capable of adapting to the tumor microenvironment (e.g., temperature, pH, and redox conditions) should be established. Among these stimuli, redox as a stimulus is especially appealing because of the great difference in the concentration of GSH between the interior (10 mM) and exterior (ca. 10 μM) of tumors [35, 36]. Furthermore, tumor tissues are highly hypoxic, with at least fourfold more GSH than normal tissues [37, 38]. Hence, the significant difference in GSH concentration can be used to remove the diffusion barrier caused by HA.

**Fig. 1:**
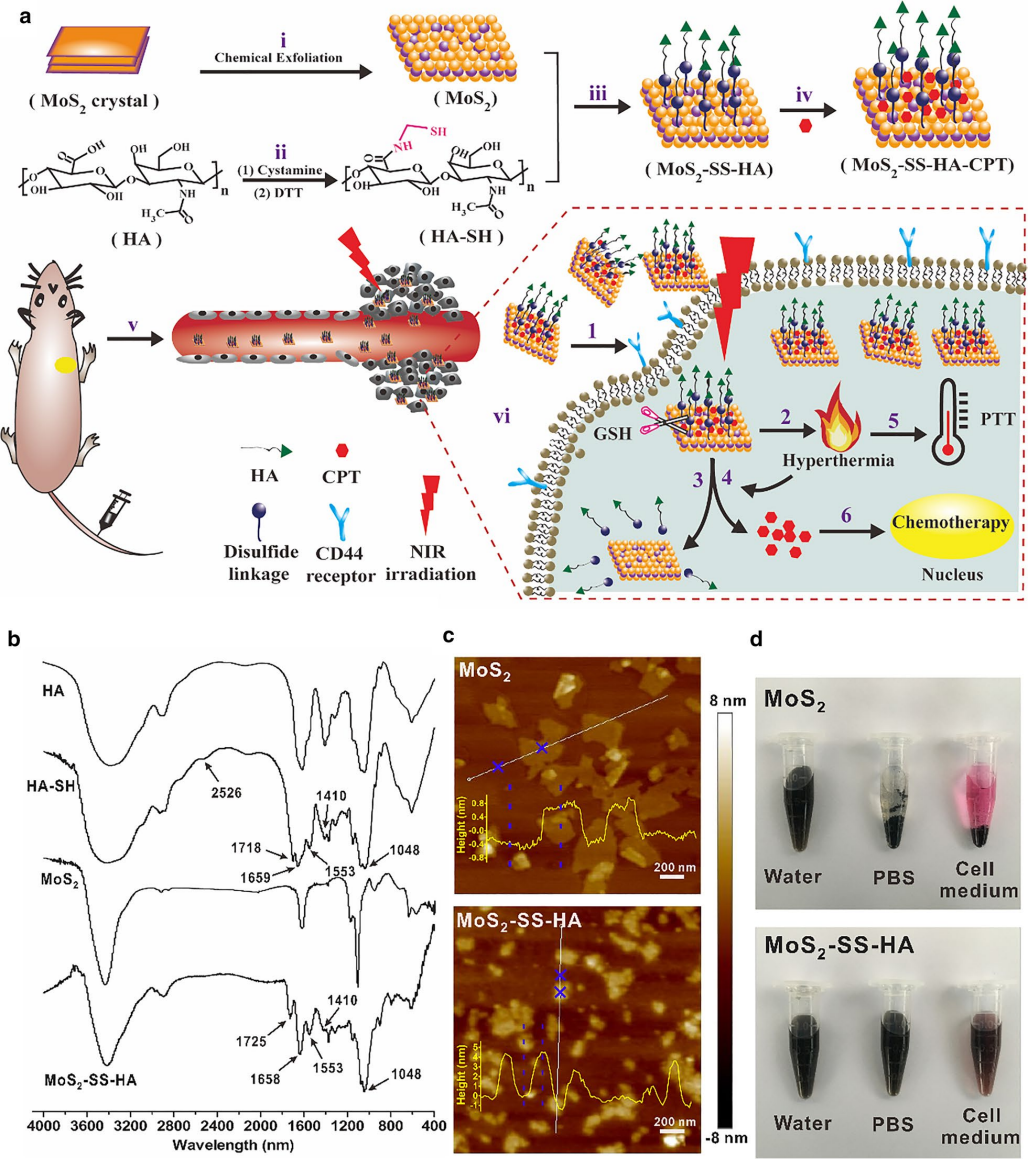
Synthesis and characterization of MoS_2_–SS–HA nanosheets. **a** Schematic of the synthesis of MoS_2_–SS–HA as a carrier capable of achieving targeted delivery of CPT and controlled drug release under dual stimuli for synergetic chemo-photothermal cancer therapy. (i, ii) Chemical exfoliation of MoS_2_ crystals to single-layered MoS_2_ nanosheets and synthesis of sulfhydrylated HA, (iii) conjugation of MoS_2_ with HA via a disulfide linkage, (iv) the CPT loading process, (v) intravenous administration of MoS_2_–SS–HA–CPT and efficient accumulation of the nanosheets at the tumor site based on passive and active targeting, and (vi) cellular uptake of HA-grafted MoS_2_ nanosheets, rapid intracellular drug release, and synergetic chemo-photothermal therapy: (1) cellular uptake of MoS_2_–SS–HA–CPT via HA-receptor-mediated endocytosis; (2) MoS_2_-based generated hyperthermia upon NIR irradiation; (3) and (4) redox- and NIR-triggered drug release; and (5) and (6) hyperthermia-induced photothermal therapy and CPT-mediated chemotherapy. **b** FT-IR spectra of MoS_2_ before and after HA coating. **c** AFM images of MoS_2_ and MoS_2_–SS–HA. **d** Stability of MoS_2_ and MoS_2_–SS–HA suspensions in water, PBS, and cell medium for 1 week

In this study, we developed a MoS_2_-based HA-functionalized drug delivery system (MoS_2_–SS–HA–CPT) that facilitated the delivery of CPT based on active targeting, responded to dual stimuli (GSH and NIR light) for rapid intracellular drug release, and achieved synergetic chemo-photothermal therapy of cancer. In brief, single-layer MoS_2_ nanosheets obtained by chemical exfoliation were conjugated with thiolated HA by forming disulfide bonds (Fig. 1a). Then, CPT, a hydrophobic broad-spectrum anticancer agent, was loaded onto the MoS_2_–SS–HA nanosheets by hydrophobic interactions, forming a drug delivery system. Cellular uptake of HA-grafted MoS_2_ nanosheets and intracellular dual-stimuli-responsive drug release were evaluated in adenocarcinoma human alveolar basal epithelial cells (A549). Finally, a series of in vitro and in vivo studies were carried out to study the synergetic chemo-photothermal therapy of cancer.

## Experimental section

### Materials

HA [molecular weight (MW) = 21 kDa] was purchased from Fureda Biological Technology Co., Ltd. (Jinan, China). MoS_2_ crystals (> 99.5%) were purchased from Muke Nano Science and Technology Co., Ltd. (Nanjing, China). CPT (> 95%), dithiothreitol (> 99%,DTT), and cystamine hydrochloride (> 96%) were acquired from Sigma-Aldrich (St Louis, MO, USA). *N*-(3-Dimethylaminopropyl)-*N*-ethylcarbodiimide hydrochloride (EDC·HCl) and *N*-hydroxysuccinimide (98%, NHS) were obtained from Aladdin Biochemical Technology Co., Ltd. (Shanghai, China). Dulbecco’s modified eagle medium (DMEM) and Kaighn’s modification of Ham’s F-12 (F-12K) medium were purchased from Life Technologies GmbH (Darmstadt, Germany). All other reagents were purchased from J&K Scientific Ltd. (Shanghai, China) and used without further purification. Amicon Ultra-15 centrifugal filters (molecular-weight cutoff (MWCO) = 100 kDa) were purchased from Millipore Corp. (Massachusetts, USA).

### Preparation of sulfhydrylated HA

Sulfhydrylated HA was synthesized according to the method reported in the literature [39]. In brief, HA (100 mg) was dissolved in 10 mL of phosphate-buffered saline (PBS, pH 7.4) followed by addition of 24 mg of EDC·HCl and 18 mg of NHS. After 1 h of reaction, the activated HA was added dropwise into 10 mL of cysteamine hydrochloride in PBS (56 mg/mL) and stirred overnight. The reaction mixture was washed with deionized water several times by ultrafiltration (3000 rpm, Sorvall ST 16R, Thermo Scientific) to remove residual EDC, NHS and cysteamine hydrochloride. Subsequently, the pH of the resulting solution was adjusted to 8.5 by addition of NaOH aqueous solution (2 M), and DTT (200 mg) was added to the reaction mixture to cleave the disulfide bond in the conjugated cystamine. After 24 h of stirring under a N_2_ atmosphere, the reaction solution was dialyzed (MWCO = 8–14 kDa) against deionized water at pH 3.5 for 2 days. Finally, the purified solution was lyophilized to obtain HA–SH as a white foam (yield 82%).

### Preparation of HA-grafted MoS_2_ via a disulfide linkage

The preparation of MoS_2_ nanosheets started from MoS_2_ crystals by the Morrison method [40]. Functionalization of the MoS_2_ with HA is quite easy to achieve because the freely exposed sulfur on MoS_2_ can easily bind to other sulfur-containing moieties by forming disulfide bonds [41, 42]. Briefly, 25 mg of HA–SH was added to 0.5 mg/mL MoS_2_ nanosheets aqueous solution (10 mL). After sonication for 30 min and stirring for 24 h, the mixture solution was dialyzed (MWCO = 100 kDa) against deionized water for 3 days to remove excess HA–SH, and the obtained MoS_2_–SS–HA was stored at 4 °C for further use.

### Characterization

Fourier transform infrared (FT-IR) spectra were obtained using a FT-IR spectrometer (Tensor 27, Bruker). Ultraviolet–visible–near infrared (UV–vis–NIR) spectra were measured using a UV–vis spectrophotometer (UV-2700, Shimadzu). Thermogravimetric analysis (TGA) was performed under an argon atmosphere using a thermal analysis system (STA449C, NETZSCH). Atomic force microscopy (AFM) images were captured using a scanning probe (Multimode 8, Bruker). Zeta-potential and dynamic light scattering (DLS) analyses were carried out using a Zetasizer (Nano ZS 90, Malvern). The temperature curves were recorded using a thermal camera (AX315, FLIR).

### Drug loading

For preparation of CPT-loaded MoS_2_ nanosheets, 2 mL of CPT in dimethyl sulfoxide (DMSO, 0.2 mg/mL) was added to an equal volume of MoS_2_–SS–HA aqueous dispersion (0.1 mg/mL). After stirring for 24 h, the excess CPT precipitated as a solid, was removed by centrifugation at 5000 rpm for 20 min. The supernatant was filtered through a 0.45-μm filter and washed several times with PBS via ultrafiltration (3000 rpm) to remove the small amount of solubilized CPT. The drug loading capacity of MoS_2_–SS–HA was determined by UV–vis spectroscopy at 365 nm.

### Dual-stimuli-responsive drug release

MoS_2_–SS–HA–CPT were enclosed in a dialysis bag and immersed in 20 mL of PBS containing DMSO (5% v/v, pH 7.4) with or without GSH, followed by placement in a shaking bed at a speed of 100 rpm. At the desired time points, a portion of the samples were irradiated under an 808-nm laser at 1 W/cm^2^ for 10 min. For each measurement, 0.2 mL of dialysis solution was removed and replaced with the same volume of the corresponding buffer. The CPT amount was determined by the absorption peak at 365 nm via UV–vis spectroscopy.

### Hemolysis assay

Female BALB/C nude mice (age, 6–8 weeks; body weight, 20–25 g) were purchased from Slac Animal Inc. (Shanghai, China). Mice were housed in clean, pathogen-free plastic cages with controlled temperature (26 °C), humidity (55%), filtered atmosphere and a 12 h light/dark cycle. Mice were provided aseptic food and water ad libitum.

Red blood cells (RBCs) from a healthy nude mouse were separated from serum by centrifugation at 2000 rpm for 10 min, washed several times with PBS, and then diluted with PBS. Various concentrations of MoS_2_–SS–HA in PBS were added to RBC solutions, which were then shaken (100 rpm) at 37 °C for 2 h. After high-speed centrifugation (15,000 rpm for 10 min), the absorbance of the supernatant from each sample at 541 nm was measured via UV–vis spectroscopy. In this experiment, solutions of RBCs mixed with deionized water and PBS alone were employed as the positive and negative controls, respectively. The hemolysis percent was calculated as hemolysis percent (%) = (A_treated_ − A_negative_)/(A_positive_ − A_negative_) × 100%.

### Cell culture

A549 and human embryonic lung fibroblasts (HELF) cells were purchased from the Cell Resource Center of Shanghai Institute for Biological Sciences (Chinese Academy of Sciences). A549 and HELF cells were cultured in F-12 K medium and DMEM medium, respectively. The medium was supplemented with 10% (v/v) fetal bovine serum (Invitrogen) and 1% penicillin/streptomycin. The cells were all maintained at 37 °C in a humidified atmosphere with 5% CO_2_.

### Cytotoxicity of MoS_2_–SS–HA in vitro

In the current work, 3-(4,5-dimethyl-2-thiazolyl)-2,5-diphenyl-2*H*-tetrazolium bromide (MTT) assays were used to investigate the cytotoxicity of MoS_2_–SS–HA in vitro. In brief, A549 and HELF cells were seeded into 96-well cell culture plates at 5 × 10^3^/well and 6 × 10^3^/well, respectively, and incubated for 24 h. The cells were washed with PBS and treated with various concentrations of the material for 48 h. After these treatments, a MTT assay was carried out to determine cell viability.

### Cellular uptake of HA-grafted MoS_2_ nanosheets

In this experiment, rhodamine B (RB) was employed as a fluorescent probe. Briefly, 1 mL of RB aqueous solution (1 mg/mL) was mixed with 4 mL of MoS_2_–SS–HA in deionized water (1 mg/mL) and stirred at room temperature for 24 h. Excess RB was removed by ultrafiltration (3000 rpm), and the resulting fluorescent-labeled MoS_2_–SS–HA was stored at 4 °C for future use.

A549 and HELF cells were seeded in confocal dishes at densities of 3 × 10^5^ and 4 × 10^5^ cells/dish, respectively, and allowed to grow for 24 h. The cells were pretreated with 0 or 5 mg/mL HA for 1 h, followed by incubation with MoS_2_–SS–HA–RB ([RB] = 40 μg/mL) for another 2 h. After the cells were stained with Hoechst 33258, fluorescence images of the cells were acquired via a confocal microscopy (TCS SP8, Leica) in the two channels relevant to Hoechst 33258 (405 nm) and RB (550 nm).

For flow cytometry analyses, A549 and HELF cells preseeded in 6-well plates at densities of 3 × 10^5^ and 4 × 10^5^ cells/well, respectively, were treated with 0 or 5 mg/mL HA for 1 h, washed with PBS, and then incubated with MoS_2_–SS–HA–RB ([RB] = 40 μg/mL) for another 2 h. After being rinsed with PBS, trypsinized, and centrifuged, the collected cells were analyzed via a flow cytometry (FACSAriaIII, BD).

### Intracellular cargo release

A549 cells were seeded in confocal dishes at a density of 3 × 10^5^ cells/dish and incubated for 24 h. The cells were treated with 0 or 10 mM glutathione reduced ethyl ester (GSH-OEt) for 2 h. Fresh cell medium containing MoS_2_–SS–HA–RB ([RB] = 40 μg/mL) was added to the dishes and replaced the original medium. After incubation for 2 h, the cells were transferred into fresh complete medium, either not irradiated or irradiated with an 808-nm laser (1 W/cm^2^) for 10 min, and then imaged via confocal microscopy.

The intracellular cargo release mechanism was also characterized by flow cytometry analyses. Briefly, A549 cells preseeded in 6-well plates at a density of 3 × 10^5^ cells/well were treated with 0 or 10 mM GSH-OE for 2 h, washed with PBS, and then incubated with MoS_2_–SS–HA–RB ([RB] = 40 μg/mL) for another 2 h. Excess nanosheets were removed, and fresh complete medium was added to the wells. After NIR irradiation (1 W/cm^2^ for 10 min), the cells were harvested with trypsin and resuspended in PBS buffer for flow cytometry analyses.

### Cytotoxicity of MoS_2_–SS–HA–CPT in vitro

The cytotoxicity of MoS_2_–SS–HA–CPT in vitro was evaluated using MTT assays. Typically, A549 cells preseeded into 96-well plates at 5 × 10^3^/well were incubated with cell medium containing 0 or 10 mM GSH-OE for 2 h. The cells were then washed with PBS and treated with MoS_2_–SS–HA–CPT or free CPT (dissolved in DMSO and diluted in cell medium) at CPT concentrations of 3.15, 6.3, 12.5, 25, and 50 μg/mL for 2 h. After this treatment, excess CPT and MoS_2_–SS–HA–CPT were removed by extensive washing with PBS, and fresh cell medium was used to culture the cells for another 48 h prior to the MTT assay to determine cell viability.

### In vitro synergistic chemo-photothermal therapy

A549 cells preseeded into 96-well plates at 5 × 10^3^/well were divided into five groups as follows: (i) culture medium as a control, (ii) culture medium + NIR, (iii) CPT + NIR, (iv) MoS_2_–SS–HA + NIR, and (v) MoS_2_–SS–HA–CPT ([CPT] = 20 μg/mL, [MoS_2_–SS–HA] = 100 μg/mL) + NIR. After treatment with 0 or 10 mM GSH-OEt for 2 h, the cells were incubated with cell medium containing the corresponding formulations. After 2 h, fresh cell medium was added to the dishes and replaced the original medium. The cells in groups ii, iii, iv, and v were irradiated with an 808-nm laser for 10 min (1 W/cm^2^). Afterwards, all of the cells were further incubated for 48 h, and the MTT assay was carried out to measure cell viability. Meanwhile, microscopic observation of the morphologies of cells after various treatments was achieved using an inverted phase-contrast microscope (BDS300, Optec).

### Tissue biodistribution of MoS_2_–SS–HA–CPT

The tissue biodistribution of MoS_2_–SS–HA–CPT was assessed in lung cancer cell-bearing nude mice. For establishment of the tumor model, A549 cells (5 × 10^6^) suspended in 50 μL PBS were subcutaneously injected into the forelimb of female BALB/C nude mice. When the mean tumor volume reached approximately 200 mm^3^, the mice were randomly divided into five groups (n = 3 per group) and intravenously administered MoS_2_–SS–HA–CPT (1.2 mg/kg) + HA (50 mg/kg) or MoS_2_–SS–HA–CPT. At set time points (6, 12, 24 and 48 h), the mice were sacrificed, and tissue samples (heart, liver, spleen, lung, kidney and tumor) were collected and weighed. The amount of MoS_2_–SS–HA–CPT in the major organs and tumor was quantified according to a method reported by Liu et al. [21]. After the tissue samples were digested by aqua regia, the Mo amount in the major organs and tumors was measured via inductively coupled plasma-atomic emission spectrometry (ICP-AES).

### In vivo synergistic chemo-photothermal therapy

The tumor model was established as described in “Tissue biodistribution of MoS_2_–SS–HA–CPT” section. When the tumor volume increased to approximately 80 mm^3^, the nude mice were randomly divided into 5 groups (n = 5/group) and intravenously injected with 200 μL of (i) PBS containing 1% DMSO (v/v) as a control, (ii) CPT (dissolved in the aforementioned PBS), (iii) MoS_2_–SS–HA + NIR, (iv) MoS_2_–SS–HA–CPT, or (v) MoS_2_–SS–HA–CPT ([CPT] = 0.2 mg/kg, [MoS_2_–SS–HA] = 1 mg/kg) + NIR. After 24 h, groups (i), (ii), (iii) and (v) were subjected to NIR irradiation under an 808-nm laser at 1 W/cm^2^ for 10 min. During the irradiation, a FLIR thermal camera was used to monitor the real-time temperature changes in tumors. The body weight and tumor size of each mouse were measured and recorded every 3 days. The tumor volume was calculated as follows: V (volume, mm^3^) = length (mm) × width^2^ (mm^2^)/2. The tumor growth inhibition ratio (R) was calculated as follows: R (%) = 100% × (V_control group_ − V_experiment group_)/V_control group_. After 24 days, all of the mice were sacrificed, and the major organs were collected for hematoxylin–eosin (HE) staining.

### Statistical analysis

Quantitative data are presented as the mean ± SD of at last three independent experiments. Statistical significance was assessed via Student’s t-test or one-way ANOVA using the GraphPad Prism 5 software. A p-value less than 0.05 was considered significant.

## Results and discussion

### Preparation and characterization of MoS_2_–SS–HA nanosheets

MoS_2_ nanosheets were synthesized from MoS_2_ crystals using a chemical exfoliation method [40]. In this case, some surface sulfur atoms of MoS_2_ were lost during exfoliation, forming defects available for binding with sulfur-containing moieties via the formation of disulfide linkages [41, 42]. In the current work, the preparation of redox-sensitive MoS_2_-based HA-functionalized nanosheets was divided into two steps: first, cystamine was conjugated via EDC/NHS coupling, and DTT was used to generate free thiol groups in the backbone of HA by cleaving the disulfide linkage in the conjugated cystamine moiety; second, the thiolated HA was coupled to the MoS_2_ nanosheets by forming disulfide bonds. The resulting MoS_2_–SS–HA nanosheets were characterized by FT-IR spectroscopy. According to the FT-IR analysis (Fig. 1b), compared with HA, HA–SH showed new absorption peaks at 1659 cm^−1^, 1553 cm^−1^, and 2526 cm^−1^ associated with the stretching vibrations of –CO–, –NH–, and –SH bonds, respectively, suggesting successful preparation of sulfhydrylated HA. After conjugation with MoS_2_, the –SH– band at 2526 cm^−1^ disappeared; however, several new absorbance peaks characteristic of HA–SH emerged at 1725 cm^−1^ (C=O stretching of –COOH), 1658 cm^−1^ (amide I band), 1553 cm^−1^ (amide II band), 1410 cm^−1^ (carboxylate symmetric stretching), and 1048 cm^−1^ (C–O stretching). The data indicated the attachment of HA–SH through the attachment of the thiol moiety to the defects of MoS_2_ by forming disulfide bonds. The presence of HA on MoS_2_ was also proven by UV–vis spectroscopy (Additional file 1: Figure S1), and the weight percentage of HA–SH in MoS_2_–SS–HA was calculated to be 20.5% by TGA (Additional file 1: Figure S2).

The morphology and size-associated properties of MoS_2_ and MoS_2_–SS–HA were investigated using AFM and DLS. As shown in the AFM images (Fig. 1c), MoS_2_ with a flat surface showed a height of ~ 1 nm, consistent with the height of MoS_2_ samples reported previously, indicating complete exfoliation of multilayered MoS_2_ to single-layered nanosheets [5]. After HA was grafted onto MoS_2_, the height of MoS_2_ increased to ~ 4 nm, suggesting the existence of HA coating. However, the size of MoS_2_ nanosheets measured by AFM decreased from ~ 420 to ~ 170 nm after HA coating because of the sonication treatment, as also proven by the DLS results (Additional file 1: Figure S3a). Because of the introduction of carboxyl groups from HA–SH, the zeta potential of MoS_2_ became highly negative (− 43 mV, Additional file 1: Figure S3b).

After confirming the successful preparation of MoS_2_–SS–HA, we investigated its stability in deionized water, PBS, and cell medium. As shown in Fig. 1d, the MoS_2_ nanosheets were stable in deionized water with no agglomeration but rapidly aggregated (within 60 min) in PBS and cell medium due to the screening of the electrostatic charge on the MoS_2_ surface [43]. In contrast, the MoS_2_–SS–HA nanosheets still dispersed well in deionized water, PBS, and cell medium after storage for 1 week. The high physiological stability provided by HA is critical for the application of MoS_2_–SS–HA nanosheets in biological fields.

### Redox-sensitive MoS_2_–SS–HA nanosheets

**Fig. 2:**
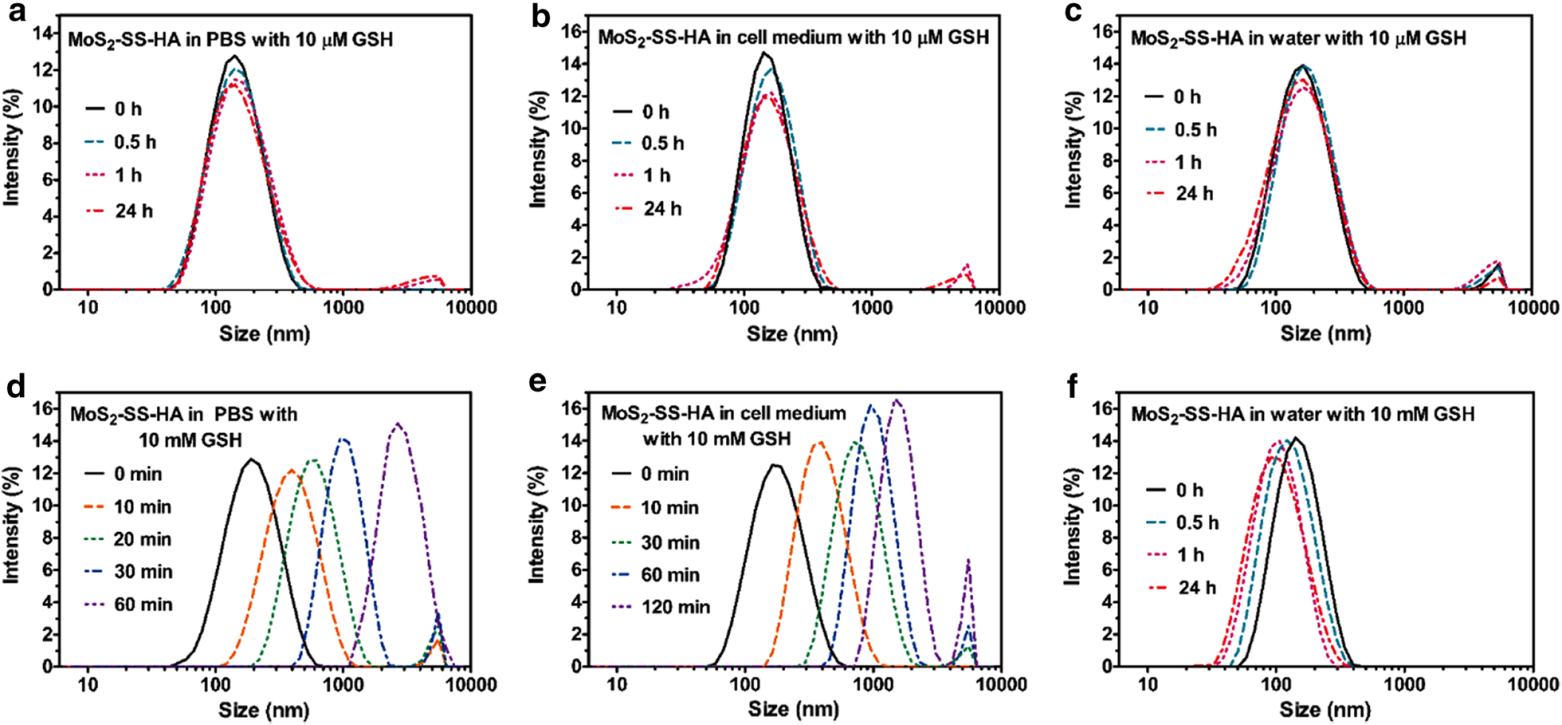
**a–f** Size-time dependence of MoS_2_–SS–HA when dispersed in PBS, cell medium, and water with different GSH concentrations, as determined by DLS measurements

The redox stimuli-responsiveness of MoS_2_–SS–HA was verified by changes in the size of the nanosheets after exposure to reducing agents (e.g., GSH). As shown in Fig. 2a–c, after 24 h of immersion in 10 μM GSH solutions (deionized water, PBS and cell medium), the MoS_2_–SS–HA maintained its structural integrity, with no change in nanosheet size. The good stability of MoS_2_–SS–HA in a physiological environment with a low concentration of a biological reducing agent was expected to decrease the leakage of encapsulated drugs during blood circulation. Upon exposure to PBS with 10 mM GSH, the MoS_2_–SS–HA exhibited a rapid increase in diameter from ~ 180 to ~ 2670 nm within 60 min (Fig. 2d). This result is attributed to cleavage of the disulfide bonds in the presence of high GSH concentrations and to separation of HA from MoS_2_, resulting in stacking of the naked MoS_2_ nanosheets. Furthermore, severe agglomeration occurred after 6 h in PBS due to the accumulation of MoS_2_. A similar phenomenon was again observed in cell medium with 10 mM GSH (Fig. 2e). By contrast, the average size of MoS_2_–SS–HA nanosheets in deionized water containing 10 mM GSH decreased from 160 to 105 nm within 1 h (Fig. 2f). These results reveal that the structural integrity of MoS_2_–SS–HA is maintained during blood circulation in vivo but is disrupted by tumor-associated GSH.

### Photothermal performance of MoS_2_–SS–HA nanosheets

**Fig. 3:**
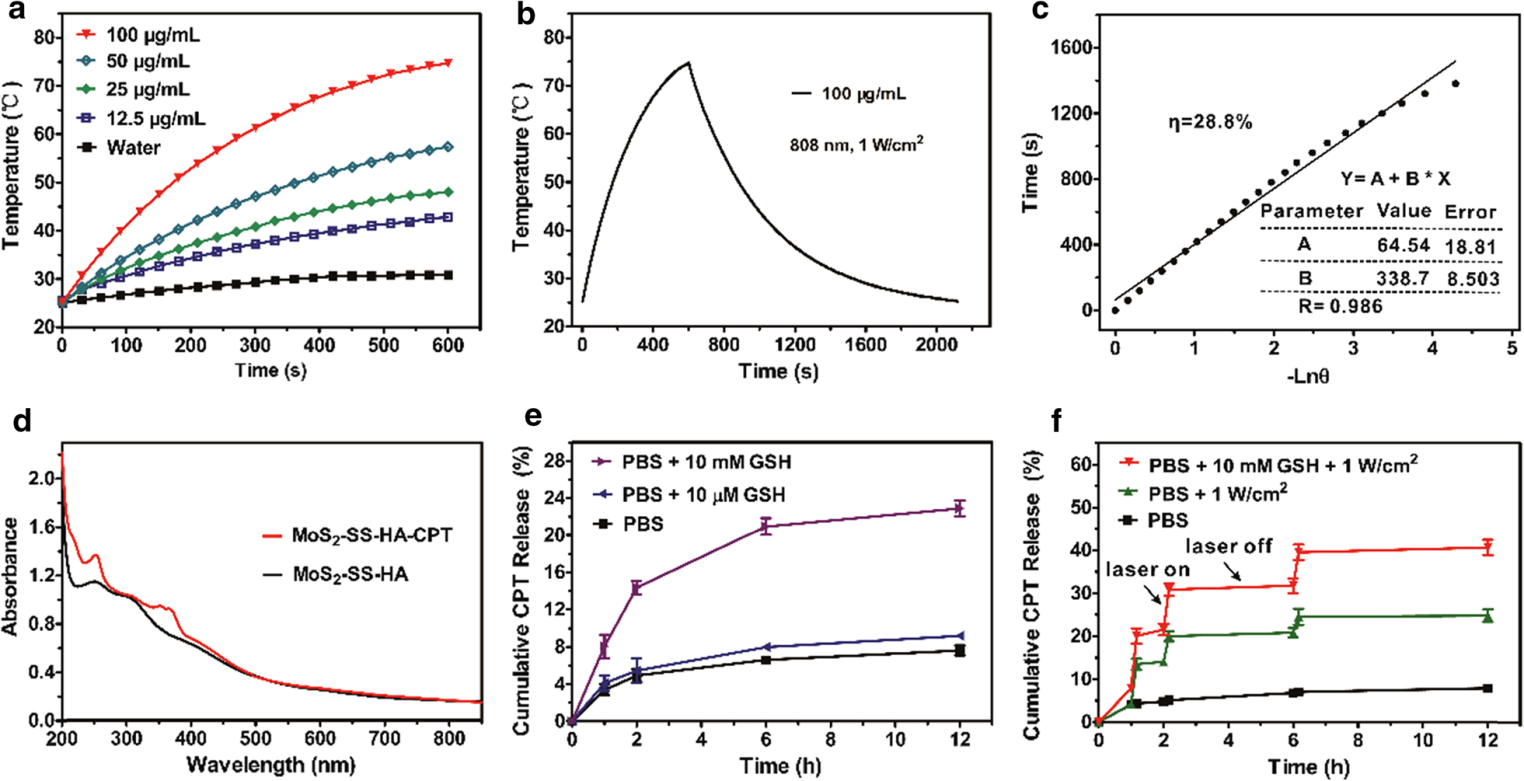
Photothermal performance of MoS_2_–SS–HA and redox/NIR light dual-stimuli-responsive drug release. **a** Temporal temperature elevation of MoS_2_–SS–HA suspensions (12.5, 25, 50, and 100 μg/mL) upon NIR irradiation (1 W/cm^2^). **b** Temperature profile of a MoS_2_–SS–HA suspension (100 μg/mL) irradiated with an 808-nm laser at 1 W/cm^2^ for 600 s, followed by natural cooling without irradiation. **c** Linear time data versus − Lnθ obtained from the cooling time of **b**. **d** UV–vis–NIR spectra of CPT-loaded MoS_2_–SS–HA nanosheets. **e**, **f** GSH- and NIR light-triggered drug release from MoS_2_–SS–HA–CPT in PBS (pH 7.4)

Photothermal therapy of cancer involves conversion of absorbed NIR light into vibrational energy by photothermal agents, which generates heat to induce tumor ablation [44]. Hence, the optical properties of MoS_2_–SS–HA must be investigated prior to application of MoS_2_–SS–HA as a photothermal agent for cancer therapy. In this study, we first examined whether the presence of HA affected the strong absorbance of MoS_2_ in a NIR region (from 700 to 850 nm). As shown in Additional file 1: Figure S1, the UV–vis–NIR spectra of MoS_2_ revealed that decoration with HA did not alter the NIR absorbance of MoS_2_. We subsequently investigated the photothermal properties of MoS_2_–SS–HA by monitoring the temperature variations of aqueous solutions containing MoS_2_–SS–HA at various concentrations (12.5, 25, 50, 100 μg/mL) under NIR irradiation (1 W/cm^2^ for 600 s). The temperature of the MoS_2_–SS–HA solutions increased rapidly during irradiation, with obvious time and concentration dependence, whereas the temperature of the water sample barely changed under the same conditions (Fig. 3a). For example, the highest temperature increment of MoS_2_–SS–HA solutions at concentrations of 12.5, 25, 50, and 100 μg/mL after NIR irradiation for 600 s was 18.1, 25.5, 33.9, and 50.1 °C, respectively. In contrast, pure water showed only a ~ 3.2 °C increase in temperature under the same irradiation conditions. Next, we measured the photothermal conversion efficiency (*ƞ*) of the MoS_2_–SS–HA nanosheets using a method reported previously [9]. Based on the obtained data (Fig. 3b, c), the *ƞ* value of MoS_2_–SS–HA nanosheets was determined to be ~ 28.8%, which was much higher than that of widely used photothermal agents, such as gold nanorods (~ 21%) and Cu_2−x_Se nanosheets (~ 22%) [45]. In addition, we studied the photothermal stability of MoS_2_–SS–HA nanosheets by recording the temperature changes of the sample with and without laser irradiation for three cycles (Additional file 1: Figure S4) and found that the temperature changes in the three cycles were consistent, indicating excellent photothermal stability of the nanosheets. This strong NIR absorbance, high photothermal conversion efficiency, and excellent photothermal stability make MoS_2_–SS–HA a highly promising photothermal agent for cancer therapy.

### Drug loading and dual-stimuli-responsive drug release

CPT is aromatic and hydrophobic, making it poorly soluble in water, which seriously diminishes its therapeutic efficacy against tumors. To address this problem, many nanosized CPT delivery systems have been developed, including GO, PLGA, and MSNs [46–48]. Unfortunately, the MoS_2_-based CPT delivery system has not been established until now. In this work, for the first time, we employed MoS_2_-based nanosheets as a carrier for the delivery of CPT. Drug loading was achieved by simply mixing CPT (dissolved in DMSO solution) with MoS_2_–SS–HA aqueous solution. After removal of unbound CPT via filtration and ultrafiltration, an absorption peak at 365 nm (characteristic of CPT) appeared in the UV–vis spectra of MoS_2_–SS–HA, offering direct evidence of CPT loading (Fig. 3d). Similar to the previously reported MoS_2_-PEG/DOX conjugates, the loading of CPT onto MoS_2_–SS–HA nanosheets was driven by hydrophobic interactions [21]. We then calculated the amount of drug loaded on the basis of the optical density of MoS_2_–SS–HA at 365 nm before and after drug loading. The CPT loading ratio (the weight ratio of the loaded drug versus MoS_2_–SS–HA) was determined to be ~ 20.6%, which is comparable to that of GO (20%) and much higher than that of PLGA (5.5%) and MSNs (1%) [46–48].

The drug release behaviors of MoS_2_–SS–HA–CPT was investigated through dialysis in PBS containing DMSO (5% v/v, pH 7.4) with or without external stimuli. A PBS buffer with different concentrations of GSH (10 μM and 10 mM) was used to simulate the extracellular and intracellular environments. As shown in Fig. 3e, less than 8% CPT was released from MoS_2_–SS–HA–CPT in PBS buffer without GSH over a period of 12 h, and the addition of 10 μM GSH to the medium triggered only a slight increase (~ 1.3%) in drug release over the same incubation time. However, upon exposure of MoS_2_–SS–HA–CPT to 10 M GSH, the cumulative release percentage of CPT rapidly increased from ~ 9.1 to ~ 22.9%. These results suggested that MoS_2_–SS–HA–CPT could not only prevent the encapsulated drug from randomly leaking during blood circulation but also trigger drug release at the tumor site based on its redox responsiveness to tumor-relevant GSH. In addition to the evaluation of GSH-induced drug release, we wondered whether MoS_2_–SS–HA–CPT possessed the same photothermal-triggered drug release property as DOX-loaded MoS_2_ nanosheets [29]. After initial NIR irradiation, the cumulative release percentage of CPT increased sharply from ~ 4.3 to ~ 13.5% within 10 min, indicating a significantly accelerated drug release process (Fig. 3f). The NIR-light-triggered drug release was ascribed to the accelerated drug molecular motion caused by the generated heat upon NIR irradiation [23]. More importantly, the cumulative drug release percentage under dual stimuli (GSH and NIR light) increased to ~ 48.1% within 12 h, which was 2.11- and 1.93-fold greater than that of GSH or NIR light alone, respectively. These findings reveal that the constructed MoS_2_ nanosheets with a unique structure can effectively prevent random leakage of the encapsulated drug during blood circulation and accelerate drug release at the tumor site under dual stimuli, thereby producing minimal side effects and maximum therapeutic efficacy.

### Biocompatibility of MoS_2_–SS–HA

**Fig. 4:**
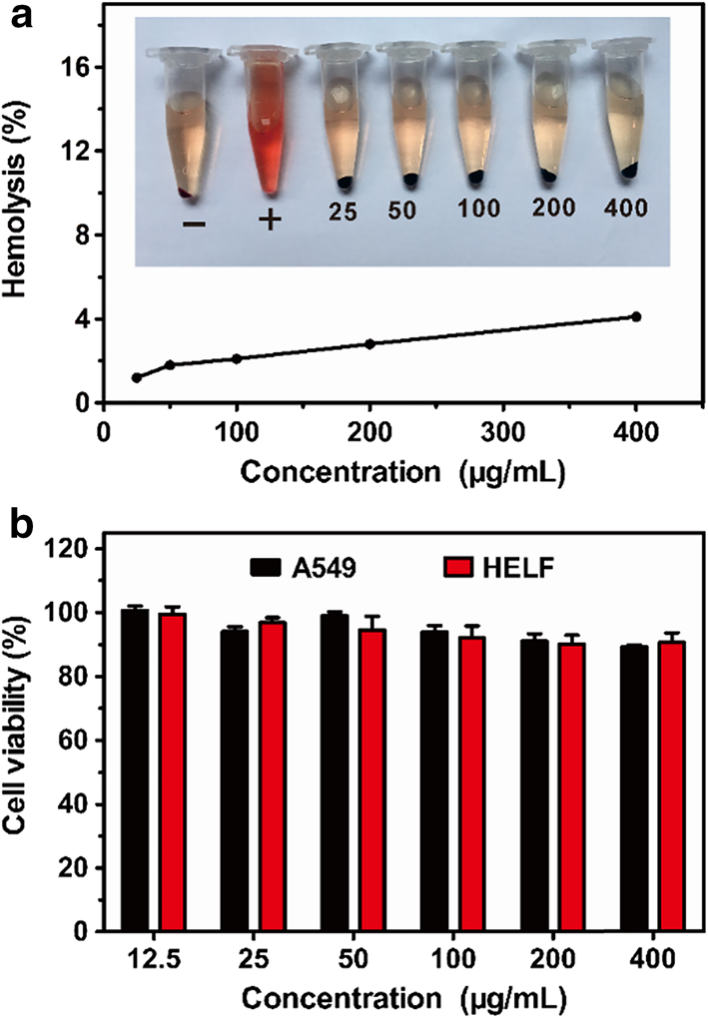
Biocompatibility of MoS_2_–SS–HA. **a** Percentages of RBC hemolysis induced by MoS_2_–SS–HA at various concentrations. Inset: images for direct observation of hemolysis. **b** Viabilities of A549 and HELF cells after incubation with cell medium containing various concentrations of MoS_2_–SS–HA for 48 h

Evaluating the biocompatibility of MoS_2_–SS–HA nanosheets is essential before their applications in biological fields. First, we assessed the blood compatibility of MoS_2_–SS–HA. After 2 h of incubation with the corresponding formulations, the optical density of all samples at 541 nm was determined by UV–vis spectrophotometry, and the hemolysis rate of MoS_2_–SS–HA with various concentrations ranging from 25 to 400 μg/mL was calculated according to the formula in “Hemolysis assay” section. As shown in Fig. 4a, no obvious hemolytic effect of MoS_2_–SS–HA was observed, with a hemolysis rate of less than 5%, demonstrating good blood compatibility of the nanosheets. In addition, we investigated the cytotoxicity of MoS_2_–SS–HA in vitro. After 48 h of incubation of A549 cells with MoS_2_–SS–HA at different concentrations, more than 90% of the A549 cells remained alive (Fig. 4b), even when the concentration of nanosheets was as high as 400 μg/mL. Moreover, no observable toxicity of MoS_2_–SS–HA to HELF cells was observed. The low cytotoxicity and negligible hemolytic effects of MoS_2_–SS–HA are conducive to their applications in the biomedical field.

### Cellular uptake and intracellular drug release

To study the cellular uptake of MoS_2_–SS–HA and the intracellular drug release kinetics by confocal microscopy and flow cytometry, the fluorescent dye RB was loaded onto MoS_2_–SS–HA to form fluorescent-labeled MoS_2_ nanosheets. The UV–vis spectra of MoS_2_–SS–HA–RB exhibited the characteristic RB peak at 575 nm, suggesting RB loading (Additional file 1: Figure S5).

**Fig. 5:**
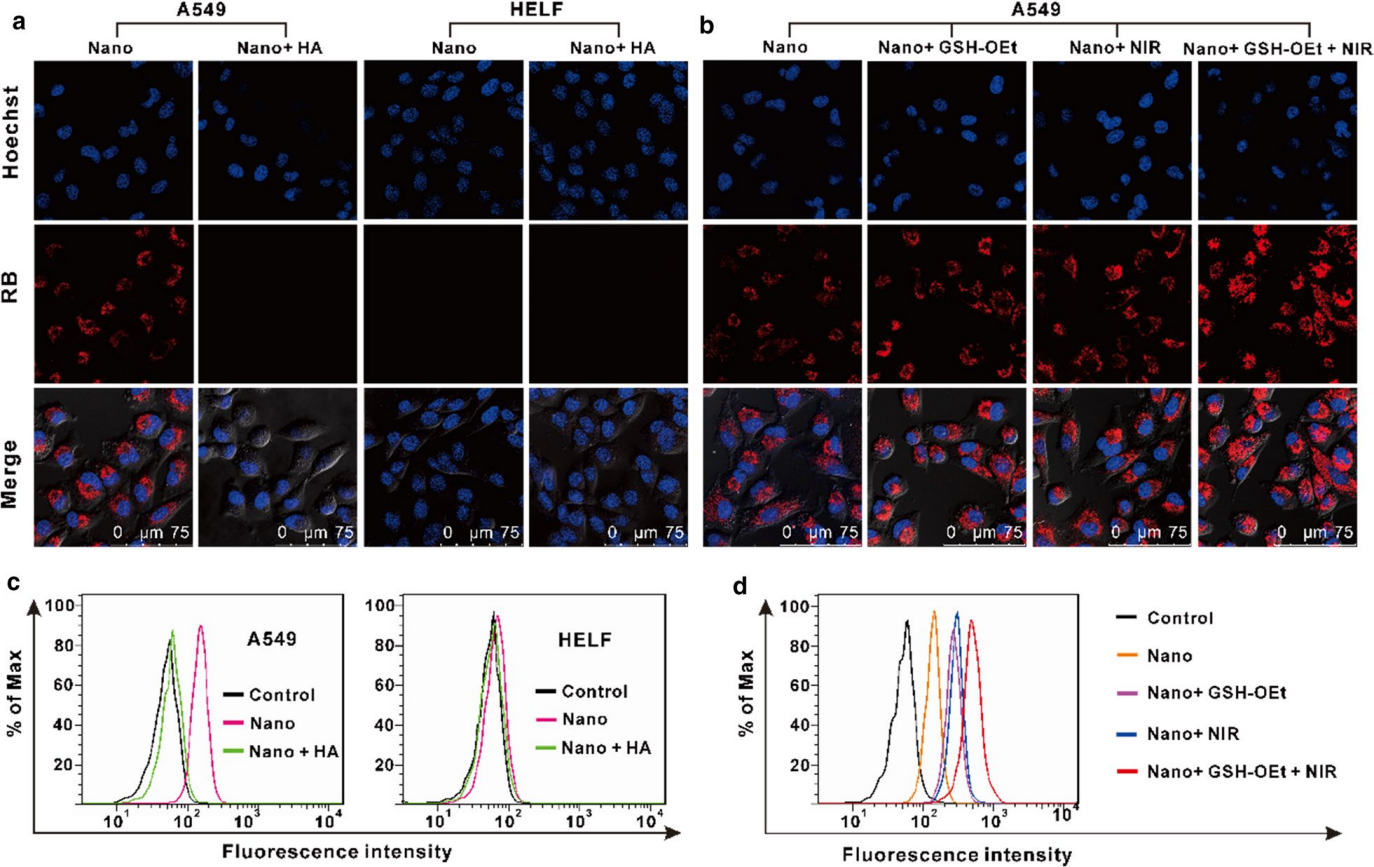
Cellular uptake of HA-grafted MoS_2_ nanosheets and intracellular cargo release. Nano: MoS_2_–SS–HA. **a** Confocal images of A549 and HELF cells incubated with MoS_2_–SS–HA–RB ([RB] = 40 μg/mL) for 2 h in the absence and presence of excess free HA. **b** Confocal images showing dual-stimuli (GSH and NIR light)-triggered intracellular cargo release. After treatment with GSH-OEt for 2 h, A549 cells were incubated with cell medium containing MoS_2_–SS–HA–RB ([RB] = 40 μg/mL) for another 2 h, washed with PBS, and irradiated with an 808-nm laser (1 W/cm^2^) for 10 min. Samples not exposed to a stimulus were used as the control. **c**, **d** Flow cytometry analysis of the intracellular fluorescence in **a** and **b**

A549 (HA receptor positive) and HELF (HA receptor negative) cells were incubated with MoS_2_–SS–HA–RB ([RB] = 40 μg/mL) for 2 h. After the cells were washing with PBS, their fluorescence images were obtained using a confocal microscope. As shown in Fig. 5a, strong RB fluorescence was observed in A549 cells, whereas HELF cells showed very weak fluorescence intensity, indicating that MoS_2_–SS–HA–RB could be transported effectively into A549 cells. To further explore the cellular uptake mechanism of HA-grafted MoS_2_ nanosheets, A549 cells were pretreated with excess HA for 1 h before incubation with MoS_2_–SS–HA–RB. As expected, a very weak fluorescence signal was detected in the HA-pretreated A549 cells, suggesting that the cellular uptake of MoS_2_–SS–HA–RB was blocked by excess HA. These results suggested that the uptake of HA-grafted MoS_2_ nanosheets by HA-receptor overexpressed cancer cells were much more efficient than that by non-cancer cells with low expression level of HA receptor, which could facilitate the accumulation of CPT delivered by the nanosheets at the tumor site.

Inspired by the results in Fig. 3e, f, we wondered whether GSH and NIR light could achieve the controlled release of the cargo inside the cells. The RB loading process caused the fluorescence quenching (Additional file 1: Figure S6), possibly due to strong interactions between the RB and MoS_2_ nanosheets [21]. From another perspective, recovery of the detected RB fluorescence may act as an indication of cargo release from the nanosheets. In this experiment, to simulate the intracellular environment, GSH-OEt, GSH-reduced ethyl ester with no cytotoxicity (Additional file 1: Figure S7), was used to increase the cellular GSH level. A549 cells were pretreated with 0 or 10 mM GSH-OEt for 2 h, followed by incubation with fresh culture medium containing MoS_2_–SS–HA–RB ([RB] = 40 μg/mL) for another 2 h. After removal of excess MoS_2_–SS–HA–RB through extensive washing with PBS, the cells were irradiated with an 808-nm laser (1 W/cm^2^) for 10 min and imaged via confocal microscopy. As shown in Fig. 5b, compared with cells not exposed to any stimulus, the MoS_2_–SS–HA–RB-incubated cells showed obviously enhanced RB fluorescence after pretreatment with GSH-OEt or NIR irradiation alone, suggesting that both GSH and NIR light could trigger the release of cargo from internalized nanosheets. The GSH-triggered intracellular cargo release was associated with the separation of the HA shell from the surface of MoS_2_ based on GSH-responsive disulfide linkage degradation. For NIR light-responsive cargo release, the MoS_2_-based generated heat upon NIR irradiation accelerated the molecular motion of the loaded cargo, resulting in rapid cargo release insides the cells. More importantly, the group of cells pretreated with GSH-OEt (10 mM) and NIR irradiation (1 W/cm^2^ for 10 min) showed the strongest RB fluorescence among the four groups, indicating that dual-stimuli-triggered intracellular cargo release was much faster than single-triggered release with only GSH or NIR irradiation.

We also studied the cellular uptake of HA-grafted MoS_2_ nanosheets and the release behaviors of cargo from the internalized nanosheets by flow cytometry. As expected, the flow cytometry data (Fig. 5c, d), consistent with the confocal imaging data, further suggested the cellular uptake of HA-grafted MoS_2_ through HA-receptor-mediated endocytosis and the rapid intracellular cargo release in response to dual stimuli.

### In vitro chemotherapy, photothermal therapy, and chemo-photothermal treatments

**Fig. 6:**
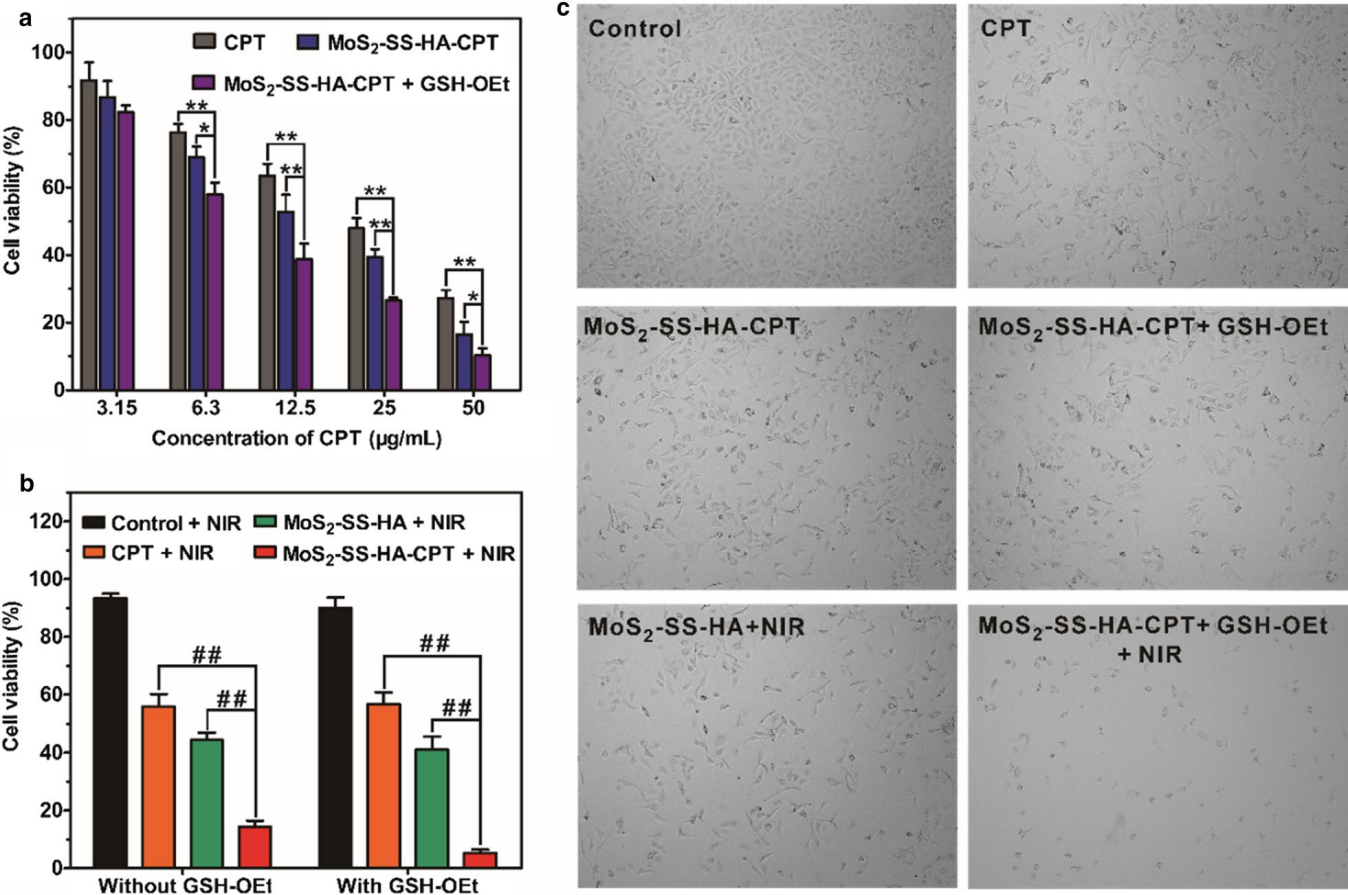
MoS_2_–SS–HA–CPT for synergetic chemo-photothermal cancer therapy in vitro. **a** Viabilities of A549 cells pretreated with GSH-OEt (0 or 10 mM) after incubation with free CPT or MoS_2_–SS–HA–CPT. A549 cells were treated with cell medium containing GSH-OEt (0 or 10 mM) for 2 h. After this treatment, the cells were incubated with free CPT or MoS_2_–SS–HA–CPT at different CPT concentrations for 2 h, washed with PBS, and then incubated for another 48 h before the MTT assay. The data are expressed as the mean ± SD (n = 5) and were analyzed via ANOVA. *p < 0.05, **p < 0.01. **b** Viabilities of GSH-OEt-treated and non-GSH-OEt-treated A549 cells incubated with free CPT, MoS_2_–SS–HA, or MoS_2_–SS–HA–CPT for 2 h, followed by NIR irradiation and incubation for an additional 48 h before the MTT assay. The data are expressed as the mean ± SD (n = 6) and were analyzed via ANOVA. ^##^p < 0.01. **c** Phase-contrast images of A549 cells after several of the treatments mentioned in **a** and **b**

The target-specific binding of HA-grafted MoS_2_ to cancer cells along with dual-stimuli-responsive drug release encouraged us to evaluate the antitumor effect of MoS_2_–SS–HA–CPT in vitro. First, A549 cells were treated with MoS_2_–SS–HA–CPT at various CPT concentrations for 2 h, washed twice with PBS, and incubated with fresh medium for another 48 h. After these treatments, the cell viability was determined using the MTT assay. As shown in Fig. 6a, both free CPT and MoS_2_–SS–HA–CPT were cytotoxic in a dose-dependent manner. Notably, MoS_2_–SS–HA–CPT exhibited higher cytotoxicity than free CPT at the same CPT concentration, possibly due to HA-targeting-enhanced cellular uptake of CPT delivered by those nanosheets. When MoS_2_–SS–HA–CPT was incubated with GSH-OEt-pretreated cells, the cytotoxicity was significantly enhanced at the tested CPT concentrations because of the rapid intracellular drug release in the presence of high GSH levels. By contrast, the toxicity of free CPT to A549 cells was not affected by the addition of GSH (Additional file 1: Figure S8).

Next, we evaluated the antitumor effect of the combined MoS_2_-induced photothermal ablation and CPT-mediated DNA damage. In this experiment, GSH-OEt-treated and non-GSH-OEt-treated A549 cells were incubated with free CPT, MoS_2_–SS–HA, or MoS_2_–SS–HA–CPT ([CPT] = 20 μg/mL, [MoS_2_–SS–HA] = 100 μg/mL). After 2 h of incubation, the cells were washed with PBS, transferred into fresh cell medium, and then irradiated with an 808-nm laser (1 W/cm^2^) for 10 min. The cell viability was determined after incubation for another 48 h. As shown in Fig. 6b, the cells treated with irradiation alone remained healthy, with ~ 93.5% cells alive. However, the viability of cells treated with MoS_2_–SS–HA–CPT was sharply reduced to ~ 13.4% after NIR irradiation. This result was attributed to the following reasons: first, the MoS_2_-based generated heat upon laser irradiation caused photothermal ablation of the cells; second, the heat accelerated the release of drug from the nanosheets, resulting in an enhanced chemotherapeutic effect. The low cell viability also suggested that synergetic chemo-photothermal therapy was more efficient in inhibiting cell proliferation than photothermal therapy (~ 44.2%) or chemotherapy (~ 55.4%) alone. When the cells were pretreated with 10 mM GSH-OEt, MoS_2_–SS–HA–CPT offered the strongest cancer-cell-killing effect under NIR irradiation, with only ~ 7% cells alive, because GSH further triggered drug release. Additionally, an inverted phase-contrast microscope was used to record the changes in cell morphology after various treatments. As expected, the morphologies of cells treated with free CPT, MoS_2_–SS–HA–CPT, MoS_2_–SS–HA–CPT + 10 mM GSH-OEt, and MoS_2_–SS–HA + NIR showed partial destruction, and the morphologies of cells treated with 10 mM GSH-OEt + MoS_2_–SS–HA–CPT + NIR were totally destroyed (Fig. 6c). These results suggest that MoS_2_–SS–HA–CPT can effectively suppress cancer cell proliferation under dual stimuli based on synergetic chemo-photothermal therapy.

### Biodistribution of MoS_2_–SS–HA–CPT in vivo

**Fig. 7:**
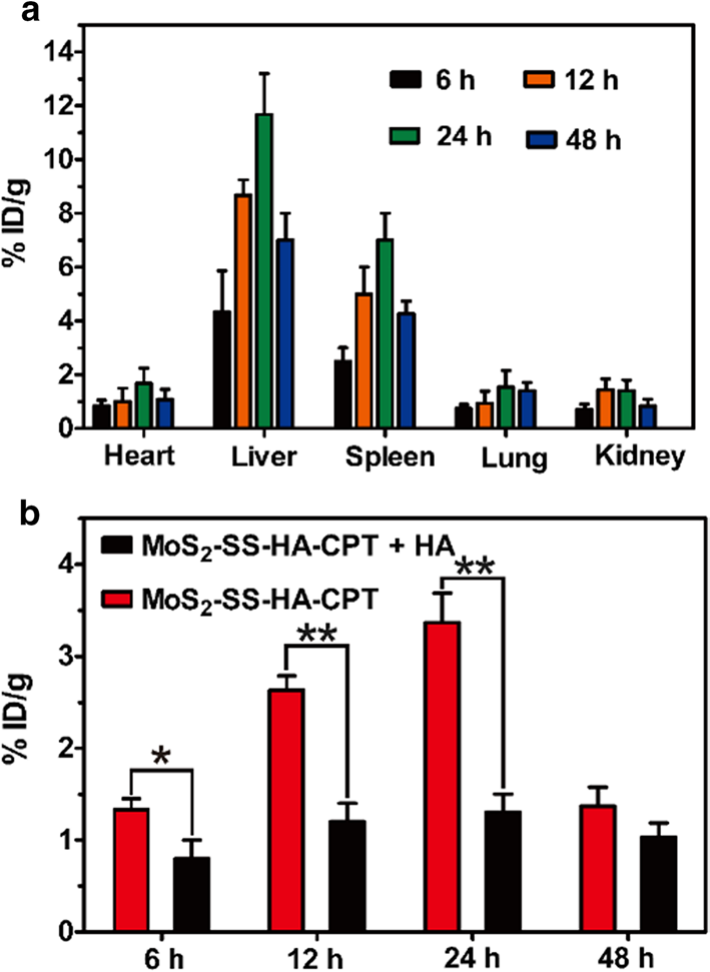
Biodistribution of MoS_2_–SS–HA–CPT in lung-cancer-cell-bearing mice. **a** Tissue biodistribution of Mo in major organs 6, 12, 24, and 48 h after intravenous administration of MoS_2_–SS–HA–CPT at a dose of 1.2 mg/kg. **b** Mo level in the tumors at 6, 12, 24, and 48 h after intravenous administration of MoS_2_–SS–HA–CPT with or without preinjection of free HA (50 mg/kg). The data are expressed as the mean ± SD (n = 3) and were analyzed by ANOVA. *p < 0.05, **p < 0.01

Tissue biodistribution of MoS_2_–SS–HA–CPT following intravenous administration was assessed by measuring the amount of Mo in major organs (liver, spleen, lung, heart, and kidney) and tumors. At designated times, mice were sacrificed, and major tissues were sliced and digested for analysis by ICP-AES. As shown in Fig. 7a, Mo was rapidly distributed into various organs at 6 h post-i.v. injection but mostly accumulated in the liver and spleen because Kupffer cells and spleen macrophages are responsible for clearing the invaders [49]. The amount of Mo in tissues reached maximum values at 24 h and decreased at later times, mainly because of the gradual clearance of MoS_2_-based nanosheets in vivo. Liu et al. reported that MoS_2_ within the physiological environment could be oxidized and transformed into water-soluble Mo(VI) oxide species (e.g., MoO_4_^2−^), which are then readily excreted from the mouse body via both renal and fecal pathways, not resulting in massive accumulation of Mo in vivo [50]. The residual Mo with a very low concentration not only did not cause side effects in vivo but also contributed to the synthesis of several enzymes in cells because Mo is an essential trace element for these enzymes [42]. To verify the tumor-targeting capacity of HA-grafted MoS_2_, free HA (50 mg/kg) was intravenously injected before administration of MoS_2_–SS–HA–CPT. We found that the Mo level in the tumors of mice treated with HA + MoS_2_–SS–HA–CPT was lower than that in tumors of mice treated with the nanosheets alone (Fig. 7b). In particular, 6, 12 and 24 h after intravenous injection, the Mo levels in the tumors of the MoS_2_–SS–HA–CPT group were 1.66-, 2.06-, and 2.43-fold higher than those in the MoS_2_–SS–HA–CPT + HA group, indicating the key role of HA in facilitating the accumulation of MoS_2_–SS–HA–CPT at the tumor site.

### In vivo chemotherapy, photothermal therapy, and chemo-photothermal treatments

**Fig. 8:**
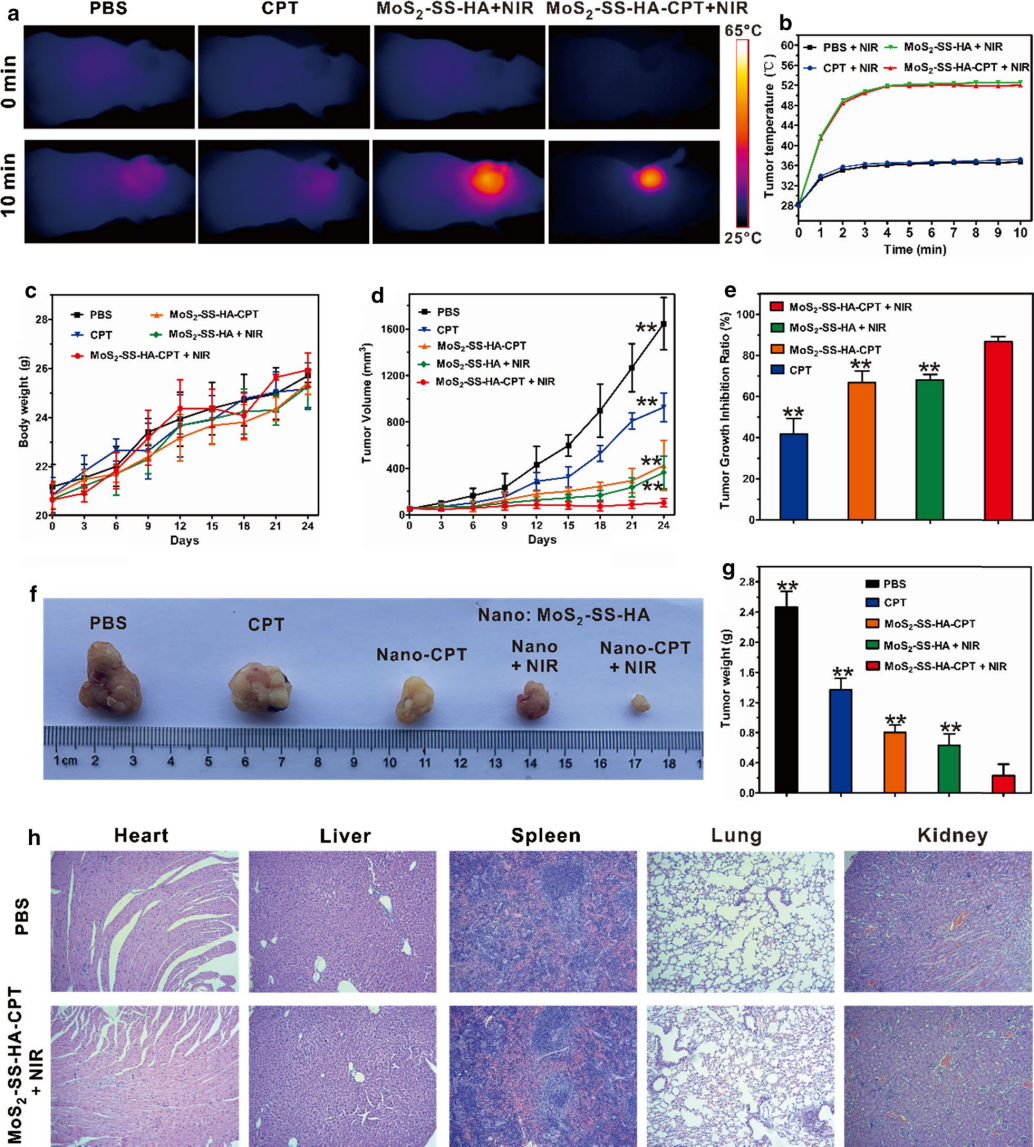
MoS_2_–SS–HA–CPT for synergetic chemo-photothermal cancer therapy in vivo. (i) PBS, (ii) CPT, (iii) MoS_2_–SS–HA + NIR, (iv) MoS_2_–SS–HA–CPT, and (v) MoS_2_–SS–HA–CPT + NIR. **a** In vivo thermal images of A549 tumor-bearing mice injected intravenously with PBS, CPT, MoS_2_–SS–HA, and MoS_2_–SS–HA–CPT under NIR irradiation. **b** Temperature changes in tumors in groups (i), (ii), (iii), and (v) during NIR irradiation. **c** Body weights of mice in each group as a function of time. **d** Tumor growth curves of each group after various treatments. Significant differences appeared between the MoS_2_–SS–HA–CPT + NIR group and the other groups and are marked as **p < 0.01. **e** Tumor growth inhibition ratio of the experimental groups. Significant differences appeared between the MoS_2_–SS–HA–CPT + NIR group and the other experimental groups and are marked as **p < 0.01. **f** Photographs of tumors collected from the five groups after 24 days. **g** Tumor weights in each group. Significant differences appeared between the MoS_2_–SS–HA–CPT + NIR group and the other groups and are marked as **p < 0.01. **h** HE images of major organs collected from mice after 24 days of intravenous administration of PBS and MoS_2_–SS–HA–CPT

The in vivo therapeutic efficiency of MoS_2_–SS–HA–CPT was assessed in A549 lung-cancer-cell-bearing mice. In this experiment, mice with a tumor volume of ~ 80 mm^3^ were randomly divided into five groups with five mice per group: (i) PBS control group, (ii) CPT group, (iii) MoS_2_–SS–HA–CPT group, (iv) MoS_2_–SS–HA + NIR group, and (v) MoS_2_–SS–HA–CPT + NIR group. At 24 h after intravenous injection of the corresponding formulations, the tumors of the control, CPT, MoS_2_–SS–HA + NIR, and MoS_2_–SS–HA–CPT + NIR groups were irradiated with an 808-nm laser at 1 W/cm^2^ for 10 min. An IR thermal camera was used to record thermal images of the mice (Fig. 8a) and monitor the temperature changes in tumors (Fig. 8b) during irradiation. For the control and CPT groups, the temperature in the tumor site increased to ~ 36.1 °C after 10 min of irradiation. By contrast, rapid temperature elevation in the tumors in the MoS_2_–SS–HA + NIR and MoS_2_–SS–HA–CPT + NIR groups was observed, mainly because of the targeted accumulation of HA-grafted MoS_2_ nanosheets in tumors and the high photothermal conversion efficiency of MoS_2_-based nanosheets. More importantly, the high temperature (~ 52 °C) reached after 3 min of NIR irradiation remained nearly constant during the next 7 min of irradiation, which could directly induce ablation of the tumor as well as accelerate drug release from MoS_2_–SS–HA–CPT [51]. During various 24-day treatments, the body weight and tumor volume of each group were recorded every 3 days. As shown in Fig. 8c, no obvious body weight loss was observed during the treatment with MoS_2_–SS–HA–CPT, suggesting the negligible side effects of the nanosheets in vivo. The tumor volume data in Fig. 8d, e showed that these formulations (CPT, MoS_2_–SS–HA–CPT, MoS_2_–SS–HA + NIR, and MoS_2_–SS–HA–CPT + NIR) could inhibit tumor growth. Compared with free CPT, MoS_2_–SS–HA–CPT showed a stronger antitumor effect, possibly due to the tumor-targeting capacity of the nanosheets and redox-responsive drug release. More importantly, after 10 min of continuous irradiation, the MoS_2_–SS–HA–CPT-treated mice exhibited the lowest tumor volume growth among the groups, and the tumor growth inhibition ratio of the nanosheets was determined to be as high as ~ 89.4%, which is 1.64-fold greater than that of our previously established GO-based HA-grafted gefitinib-loaded nanosheets with redox-responsive drug release [52], suggesting the excellent antitumor effect of MoS_2_–SS–HA–CPT under NIR irradiation. This result was ascribed to the MoS_2_-generated hyperthermia upon NIR irradiation that not only further facilitated the release of CPT and enhanced the chemotherapeutic efficiency of MoS_2_–SS–HA–CPT but also induced photothermal ablation of the tumor, enabling synergistic chemo-photothermal therapy and effectively inhibiting tumor growth in vivo. After 24 days, all of the mice were sacrificed and the tumors were collected, as shown in Fig. 8f. After the tumors from each group were weighed, we found that the mean tumor weight in the MoS_2_–SS–HA–CPT + NIR group was the lowest among the five groups (Fig. 8g), further indicating that MoS_2_–SS–HA–CPT could effectively inhibit tumor growth under NIR irradiation. Finally, the major organs of groups (i) and (v) were excised for histological analysis. No obvious organ damage was observed (Fig. 8h), preliminarily suggesting the safety of these nanosheets in vivo at the tested dose. As a result, the excellent antitumor effect of MoS_2_–SS–HA–CPT, without obvious side effects or organ damage, may considerably promote their future application for treatment of cancer.

## Conclusions

In summary, MoS_2_ nanosheets decorated with HA were developed as a carrier for targeted delivery of the anticancer drug CPT. Grafting of HA onto the surface of MoS_2_ via a disulfide linkage not only enhanced the stability of MoS_2_ nanosheets in a physiological environment but also facilitated release of the encapsulated drug through a GSH-mediated redox response. Moreover, the MoS_2_-based generated heat upon NIR irradiation further triggered the release of drug from MoS_2_–SS–HA–CPT and induced photothermal ablation of tumors. Confocal fluorescence images and flow cytometry data together suggested the efficient uptake of HA-grafted MoS_2_ nanosheets by A549 cells via HA-receptor-mediated endocytosis and the rapid intracellular drug release in response to dual stimuli (GSH and NIR light). Compared with other treatment groups, MoS_2_–SS–HA–CPT upon dual stimuli more effectively suppressed cell proliferation in vitro and inhibited tumor growth in vivo. The as-prepared MoS_2_–SS–HA–CPT with a strong targeting ability, dual-stimuli-responsive drug release, and synergistic cancer therapeutic efficiency may provide a new strategy for cancer therapy.

## Additional file

**Additional file 1: Figure S1.** UV–vis–NIR spectra of MoS_2_ before and after HA coating. **Figure S2.** Weight loss curves of MoS_2_-SS-HA. **Figure S3. (a)** Size and **(b)** zeta potential data of MoS_2_ before and after HA coating. **Figure S4.** Temperature variation of MoS_2_-SS-HA suspension (100 μg/mL) over 3 cycles of NIR irradiation (1 W/cm^2^) and natural cooling. **Figure S5.** UV–vis–NIR spectra of MoS_2_-SS-HA before and after RB loading. **Figure S6.** Fluorescence spectra of free RB and MoS_2_-SS-HA-RB at the same RB concentration (6 μg/mL, λex = 550 nm). **Figure S7.** Viabilities of A549 and HELF cells incubated with cell medium containing various concentrations of GSH-OEt for 48 h. **Figure S8.** Viabilities of GSH-OEt-treated A549 cells after treatment with various concentrations of free CPT.

## Data Availability

All data generated or analyzed during this study are included in this article and its additional file.
