# Supplementary material for: Redox/NIR dual-responsive MoS2 for synergetic chemo-photothermal therapy of cancer
Source: J Nanobiotechnology. 2019 Jul 3;17:78. doi: 10.1186/s12951-019-0510-2 (PMC6607525; doi:10.1186/s12951-019-0510-2)
Supplement: Supplementary file 1 — Additional file 1: Figure S1. UV–vis–NIR spectra of MoS2 before and after HA coating. Figure S2. Weight loss curves of MoS2-SS-HA. Figure S3. (a) Size and (b) zeta potential data of MoS2 before and after HA coating. Figure S4. Temperature variation of MoS2-SS-HA suspension (100 μg/mL) over 3 cycles of NIR irradiation (1 W/cm2) and natural cooling. Figure S5. UV–vis–NIR spectra of MoS2-SS-HA before and after RB loading. Figure S6. Fluorescence spectra of free RB and MoS2-SS-HA-RB at the same RB concentration (6 μg/mL, λex = 550 nm). Figure S7. Viabilities of A549 and HELF cells incubated with cell medium containing various concentrations of GSH-OEt for 48 h. Figure S8. Viabilities of GSH-OEt-treated A549 cells after treatment with various concentrations of free CPT. [file 12951_2019_510_MOESM1_ESM.docx]

**Redox/NIR dual-responsive MoS_2_ for synergetic chemo-photothermal therapy of cancer**


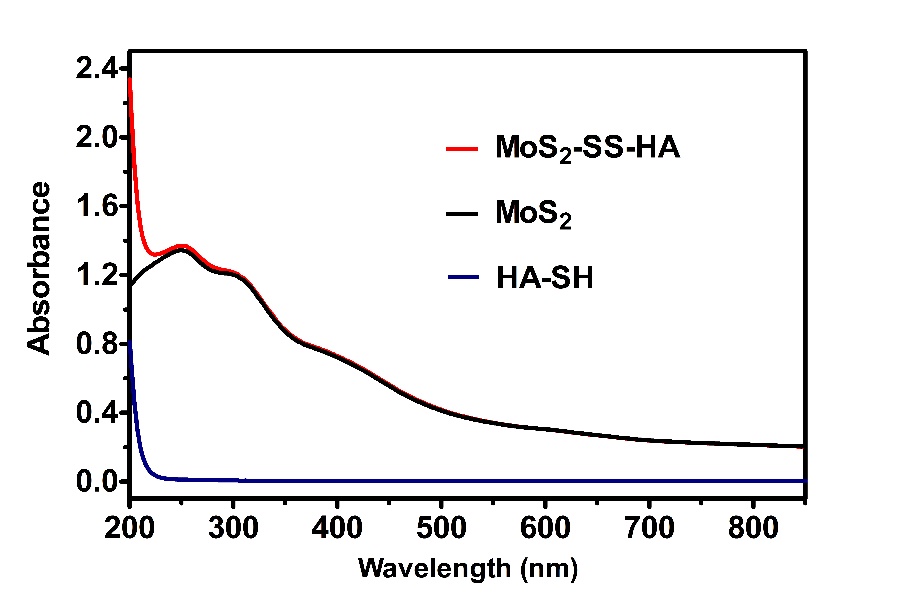


**Figure S1.** UV–vis–NIR spectra of MoS_2_ before and after HA coating. After modification of MoS_2_ with HA, the optical density of MoS_2_-SS-HA from 250 to 200 nm was higher compared with MoS_2_, due to the strong UV absorbance of HA-SH in this region, suggesting the successful preparation of MoS_2_-SS-HA.


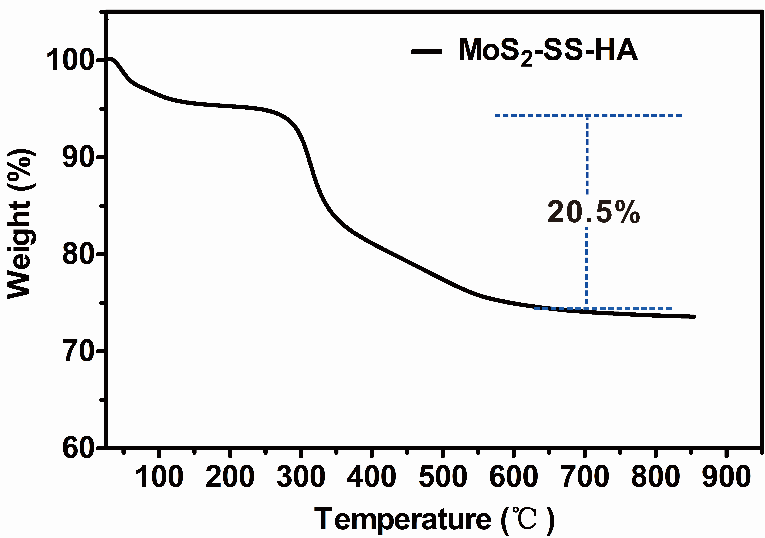


**Figure S2.** Weight loss curves of MoS_2_-SS-HA. TGA was performed on MoS_2_-SS-HA in the temperature range of 25–850°C under an argon atmosphere. A small mass loss occurred below 150°C due to the absorbed/stored water molecules. Then, a significant mass loss in the range of mainly was associated with the complete degradation of HA-SH. The weight content of HA-SH on MoS_2_ was determined to be approximately 20.5%.


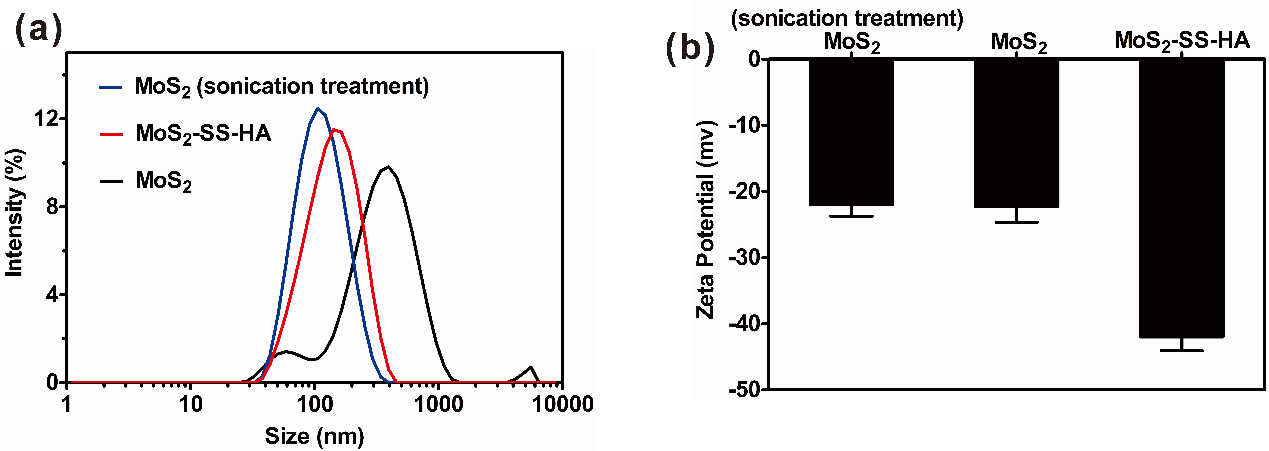


**Figure S3. (a)** Size and **(b)** zeta potential data of MoS_2_ before and after HA coating.


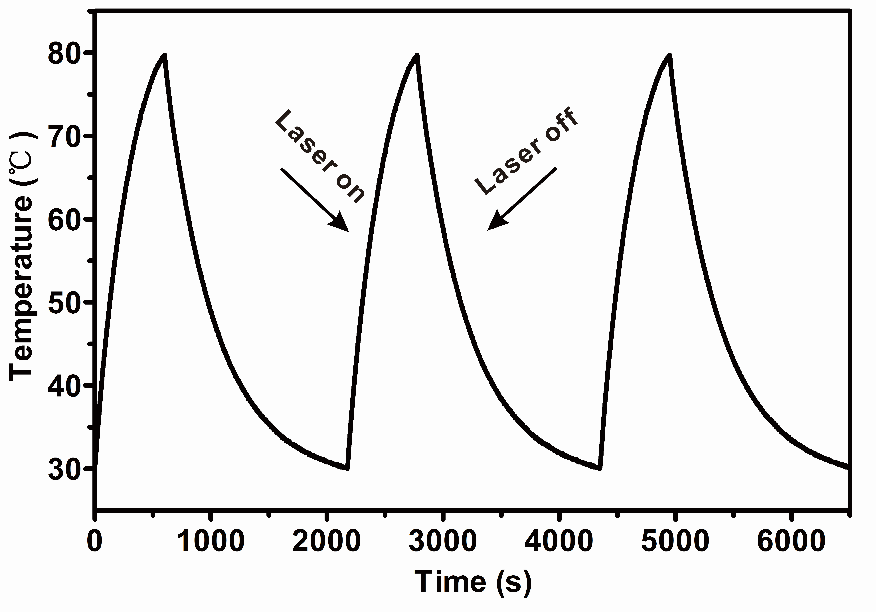


**Figure S4**. Temperature variation of MoS_2_-SS-HA suspension (100 μg/mL) over 3 cycles of NIR irradiation (1 W/cm^2^) and natural cooling.


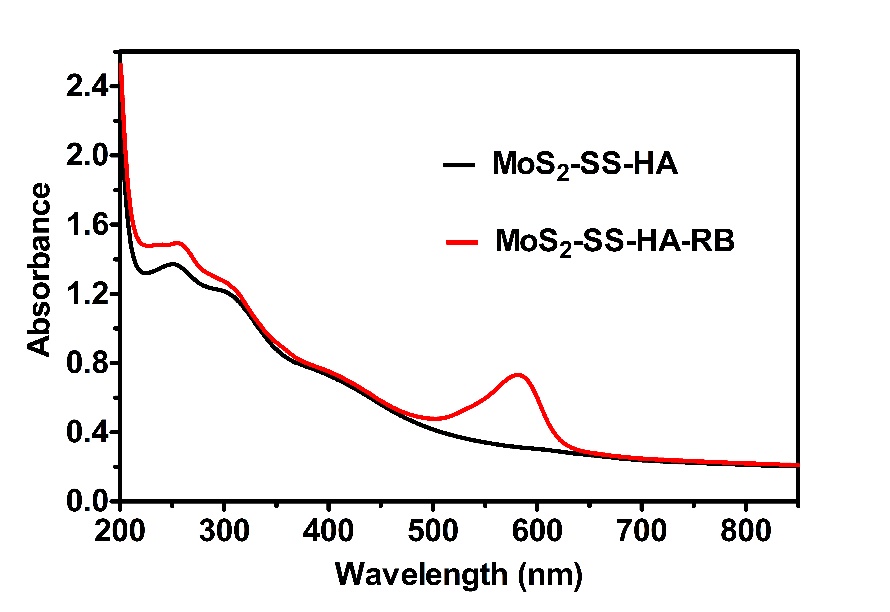


**Figure S5.** UV-vis-NIR spectra of MoS_2_-SS-HA before and after RB loading


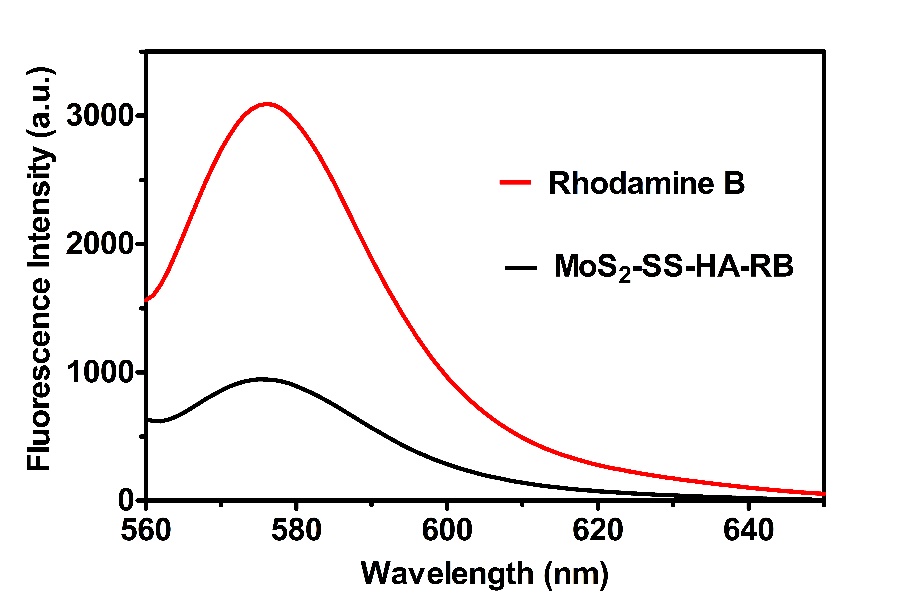


**Figure S6.** Fluorescence spectra of free RB and MoS_2_-SS-HA-RB at the same RB concentration (6 μg/mL, λex = 550 nm).


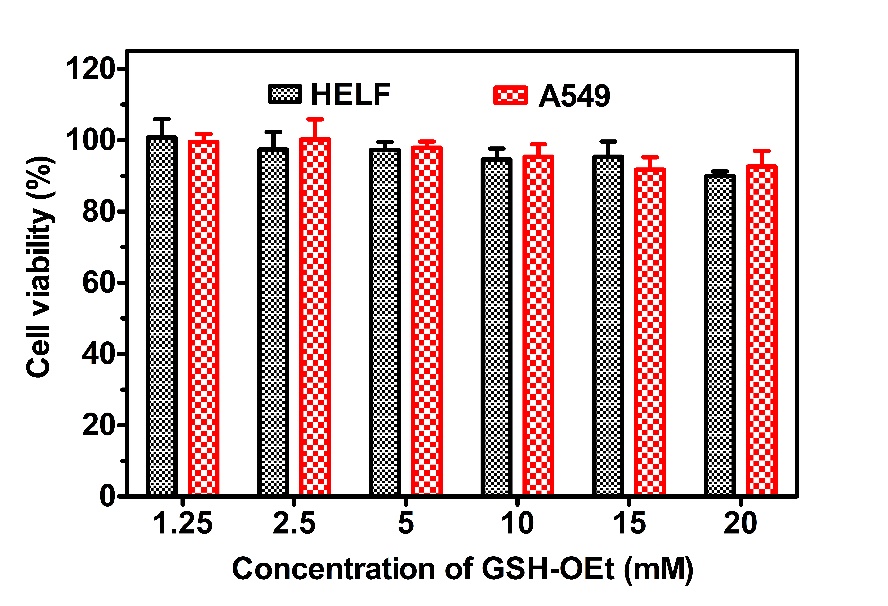


**Figure S7.** Viabilities of A549 and HELF cells incubated with cell medium containing various concentrations of GSH-OEt for 48 h.


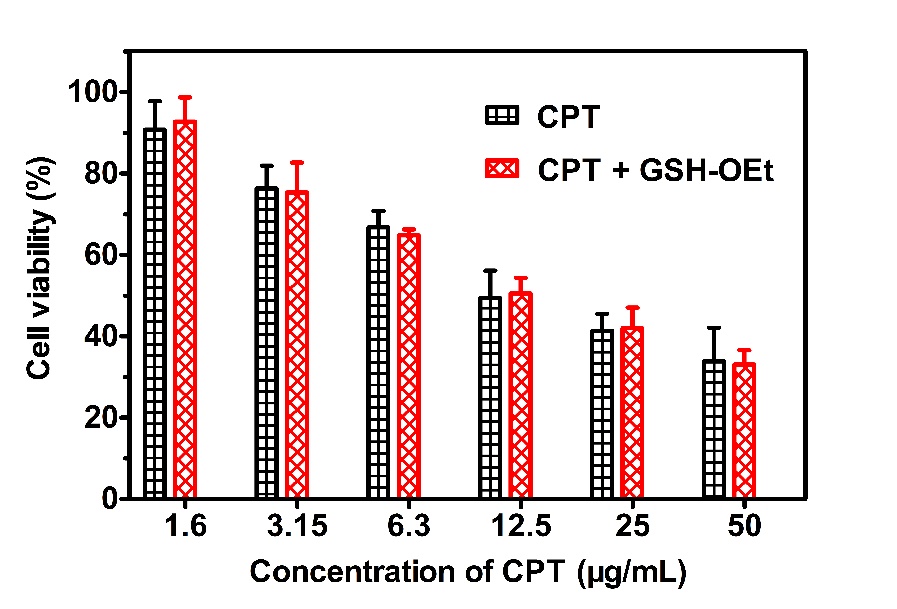


**Figure S8.** Viabilities of GSH-OEt-treated A549 cells after treatment with various concentrations of free CPT. A549 cells were treated with 10 mM GSH-OEt for 2 h and incubated with free CPT for another 2 h. After washing with PBS, free cell medium was used to culture the cells for 48 h prior to the MTT assay to determine the cell viability.
